# A comparaison of clinical diagnostic classification criteria used in longitudinal cohort studies of the Alzheimer's disease continuum: a systematic review

**DOI:** 10.1002/alz70860_101123

**Published:** 2025-12-23

**Authors:** Marie‐Jeanne Kergoat, Juan‐Manuel Villalpando, Bernard‐Simon Leclerc, Minh Tri Le, Carol Hudon, Aline Bolduc

**Affiliations:** ^1^ Faculte de medecine, Universite de Montreal, Montreal, QC, Canada; ^2^ Centre de recherche Institut universitaire de geriatrie de Montreal, Montreal, QC, Canada; ^3^ Ecole de sante publique, Universite de Montreal, Montreal, QC, Canada; ^4^ Université Laval, Quebec, QC, Canada; ^5^ CERVO Research Centre, Québec, QC, Canada; ^6^ Centre de recherche Institut universitaire de gériatrie de Montreal, Centre integre universitaire de sante et de services sociaux du Centre‐Sud‐de‐L'île‐de‐Montreal, Montreal, QC, Canada

## Abstract

**Background:**

Alzheimer's is a progressive disease, with a long preclinical phase of many decades. The current shift towards a biological, i.e., biomarker based or clinical‐biological definition of Alzheimer's disease is a fundamental conceptual change in the diagnosis of AD, one which will help correct this significant risk for misdiagnosis going forward. However, most of the ongoing or recently completed cohort studies still rely on clinical criteria or methodologies, which raise challenges when comparing data across research cohorts.

**Objective:**

A systematic review was conducted to identify and compare the diagnostic criteria used in prospective population study cohorts centering on the Alzheimer's disease clinical continuum in older adults.

**Methods:**

A review was performed of cohort studies started in the year 2000 or later, with a follow‐up duration of at least 3 years among people aged between 50‐ and 85‐years old living in the community. Original studies were searched in MEDLINE, Embase, Cochrane, PsycINFO, and Web of Science. Data were extracted from each included study using a standardized extraction form. The methodological quality of selected studies was evaluated using the National Institutes of Health (NIH) Quality Assessment Tool for Observational Cohort and Cross‐Sectional Studies.

**Results:**

Among 3297 individual papers that were eligible studies, two independent reviewers agreed on the final selection of 28 studies covering 25 cohorts. In general, the studies followed fewer than 1,500 participants. The results showed convergence in the choice of diagnostic classification criteria among the 25 cohorts studied especially for the later stages of AD, while criteria for the earliest stages showed greater variability. Only five cohorts studied were concerned with the follow‐up of the full spectrum of the disease.

**Conclusion:**

While the study of Alzheimer's disease is shifting towards a biomarker‐based diagnosis, the use of clinical diagnostic criteria for its different stages will still be a useful tool, especially for large cohorts and studies carried out in developing countries. Our review emphasizes the need of adopting a common set of clinical diagnostic criteria, specifically designed for large‐scale population studies, and shows certain areas of convergence and divergence that could aid in their development.

